# Successful destination therapy after stepwise mechanical circulatory support in a patient with cardiogenic shock following mechanical aortic valve replacement

**DOI:** 10.1007/s10047-026-01573-8

**Published:** 2026-07-31

**Authors:** Rina Ebe, Tatsuki Fujiwara, Eiki Nagaoka, Takuya Kawabata, Hironobu Sakurai, Junya Nabeshima, Takuro Takashima, Taishi Yonetsu, Makoto Araki, Yoshitaka Isotani, Tetsuo Sasano, Tomoyuki Fujita

**Affiliations:** 1 Department of Cardiovascular Surgery, Institute of Science Tokyo, 1-5-45 Yushima, Bunkyo-ku, Tokyo, 113-8519 Japan; 2 Department of Cardiology, Institute of Science Tokyo, 1-5-45 Yushima, Bunkyo-ku, Tokyo, 113-8519 Japan

**Keywords:** Cardiogenic shock, Mechanical circulatory support, Impella 5.5, Left ventricular assist device, Mechanical aortic valve replacement

## Abstract

Patients with mechanical aortic valve replacement and end-stage heart failure present major challenges when mechanical circulatory support is required, as transvalvular devices such as the Impella 5.5 are contraindicated. We report a 57-year-old man with idiopathic dilated cardiomyopathy who developed cardiogenic shock after prior mechanical aortic valve replacement. Despite cardiac resynchronization therapy defibrillator support and intra-aortic balloon pumping, he became dialysis dependent. The patient’s condition progressively deteriorated, necessitating more robust mechanical circulatory support. Although durable left ventricular assist device implantation was considered, transvalvular Impella 5.5 support was precluded by the mechanical aortic valve, and simultaneous durable left ventricular assist device implantation with redo aortic valve replacement was considered high risk because of poor surgical tolerance and end-organ dysfunction. Therefore, we planned a staged surgical strategy. Reoperative aortic valve replacement with a bioprosthetic valve was therefore performed to enable Impella 5.5 implantation. Impella support resulted in hemodynamic stabilization, end-organ recovery, and improvement in risk stratification. Subsequently, a HeartMate 3 was successfully implanted as destination therapy. The patient recovered progressively and was discharged. This case demonstrates that intentional conversion to a bioprosthetic valve may be a viable strategy to enable advanced mechanical circulatory support.

## Introduction

Patients with a history of mechanical aortic valve replacement (AVR) who later progress to end-stage heart failure (HF) present unique challenges when mechanical circulatory support (MCS) is required [1]. Mechanical prosthetic valves preclude the use of transvalvular axial-flow pumps such as the Impella because leaflet interference may cause device malfunction or damage [2]. Consequently, additional surgical intervention is often required before advanced MCS can be applied.

Although mechanical valves are commonly selected in younger patients because of their durability, this advantage may become a limitation in those who later develop advanced HF. As long-term survival after mechanical AVR improves, strategies for managing post-AVR HF requiring MCS are increasingly needed. However, the optimal approach for valve reintervention to enable temporary MCS remains undefined.

We report a case of refractory cardiogenic shock after mechanical AVR successfully managed with a staged strategy consisting of redo AVR with a bioprosthetic valve, Impella 5.5 support, and subsequent HeartMate 3 implantation.

## Case report

A 57-year-old man with idiopathic dilated cardiomyopathy underwent mechanical AVR for severe aortic regurgitation and tricuspid annuloplasty at age 49. Before the first AVR, the Brain Natriuretic Peptide (BNP) level was 2231.1 pg/mL, and transthoracic echocardiography showed a markedly reduced Left Ventricular Ejection Fraction of 24.2%, Left Ventricular End-Diastolic Diameter of 68.9 mm, Left Ventricular End-Systolic Diameter of 58.4 mm, severe aortic regurgitation, and mild tricuspid regurgitation with a Tricuspid Regurgitant Pressure Gradient of 24.2 mmHg. Invasive hemodynamic assessment by cardiac catheterization was not performed at that time. One year later, cardiac resynchronization therapy defibrillator implantation was performed for severe left ventricular (LV) dysfunction. Despite optimal therapy, he developed recurrent decompensated HF and became inotrope dependent.

On admission, he required continuous intravenous dobutamine, milrinone, and furosemide. He was hypotensive (82/48 mmHg) with laboratory evidence of multiorgan dysfunction, including elevated creatinine (1.97 mg/dL), total bilirubin (3.6 mg/dL), and brain natriuretic peptide (4837 pg/mL).

Echocardiography showed severe LV dilation (end-diastolic diameter 85 mm), reduced ejection fraction (23%), severe functional mitral regurgitation, moderate-to-severe tricuspid regurgitation, and a normally functioning mechanical aortic valve. Right heart catheterization demonstrated pulmonary hypertension (61/29 mmHg, mean 41 mmHg) and a low cardiac index (2.06 L/min/m^2^).

Despite intra-aortic balloon pumping, renal function deteriorated, necessitating continuous hemodiafiltration. Because transvalvular MCS was not feasible with the mechanical valve, a two-stage strategy was planned: redo AVR with a bioprosthetic valve to permit Impella 5.5 insertion, followed by hemodynamic stabilization and eventual durable left ventricular assist device (LVAD) implantation.

**Fig. 1:**
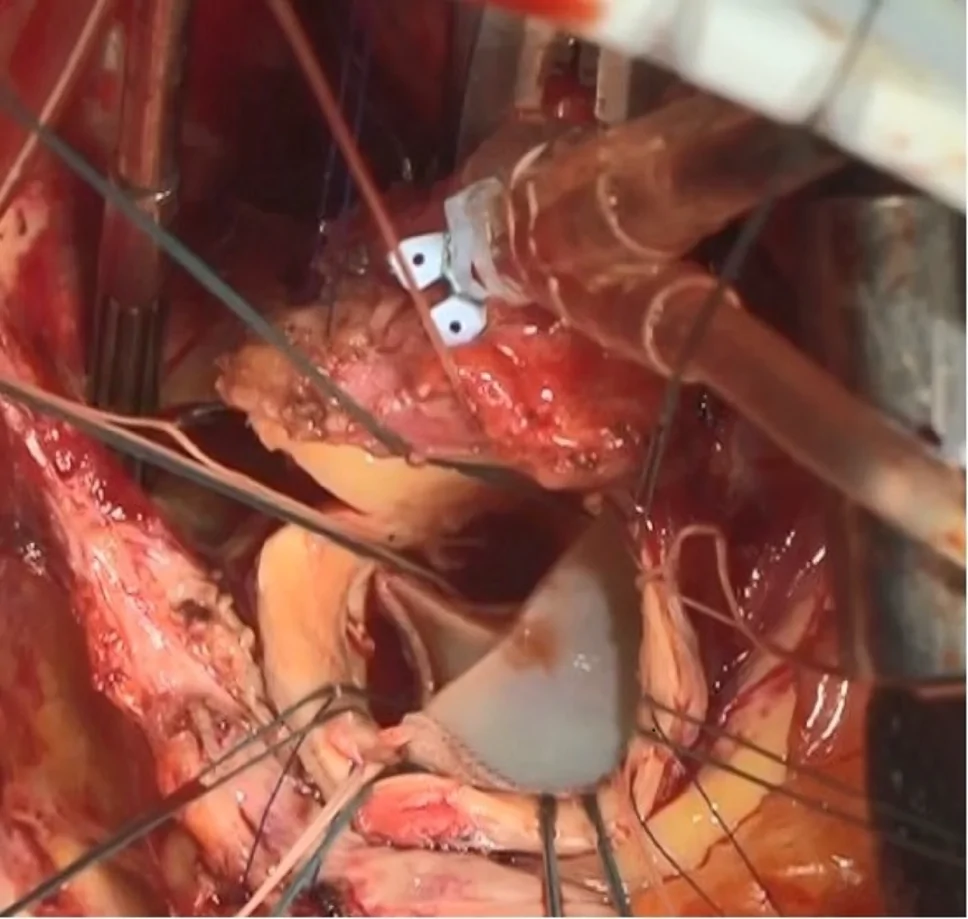
Implantation of a 23-mm INSPIRIS bioprosthetic valve

**Fig. 2:**
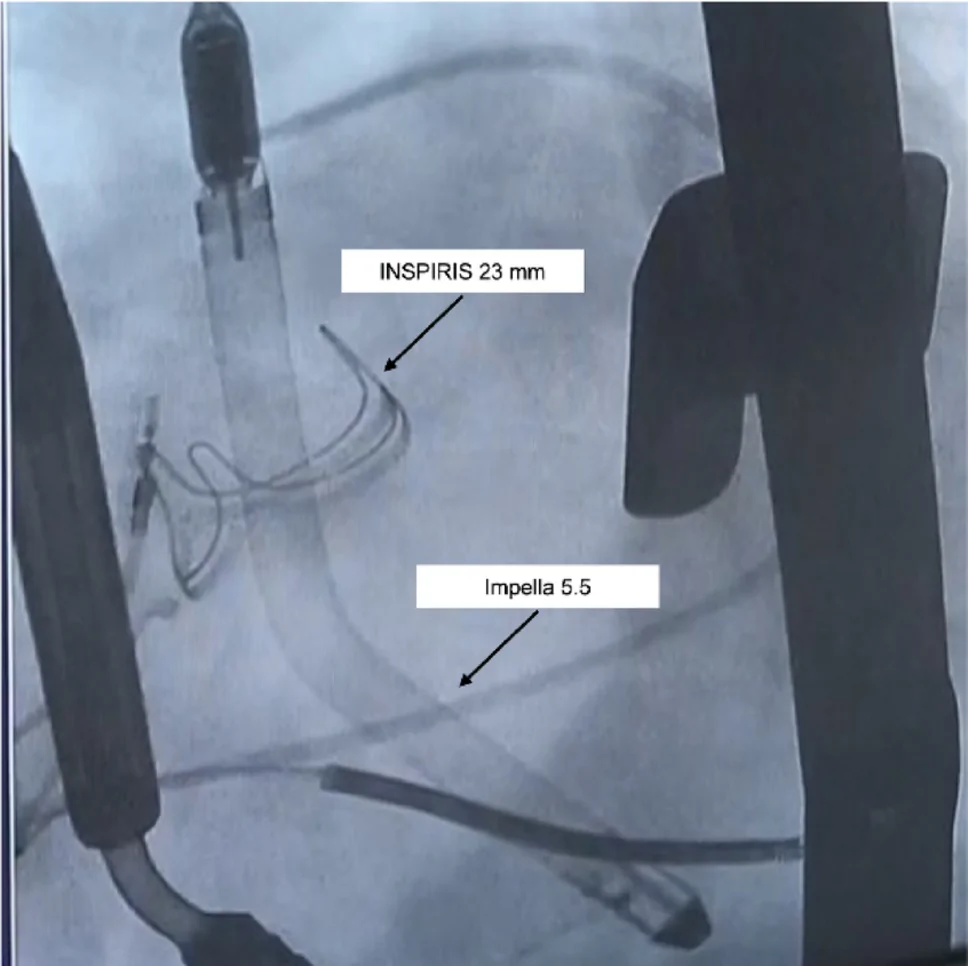
Insertion of an Impella 5.5 device across the bioprosthetic valve

After the femoro-femoral bypass was established, re-sternotomy was performed uneventfully and redo AVR was performed using a 23-mm Inspiris Resilia bioprosthesis (Fig. 1). After closing the aortotomy and removing the aortic cross-clamp, an Impella 5.5 was positioned across the bioprosthetic valve under C-arm fluoroscopic guidance (Fig. 2). The total cardiopulmonary bypass time was 282 min and the aortic cross-clamp time was 105 min. Postoperative recovery was favorable. Renal replacement therapy was discontinued on postoperative day 2, hepatic function improved, and the patient was extubated and maintained on reduced inotrope support. In parallel, the HeartMate 3 risk score decreased from 3.60 to 1.60 and the J-MACS score from 17.6 to 11.5, reflecting improved but intermediate surgical risk for durable LVAD implantation [3, 4]. These improvements collectively indicated that the patient had recovered sufficiently to safely proceed with HeartMate 3 implantation. The decision to proceed on postoperative day 11 was further informed by the aim to limit the duration of temporary MCS and its associated risks. The postoperative course following HeartMate 3 implantation was uneventful, without recurrence of renal or hepatic dysfunction.

**Fig. 3:**
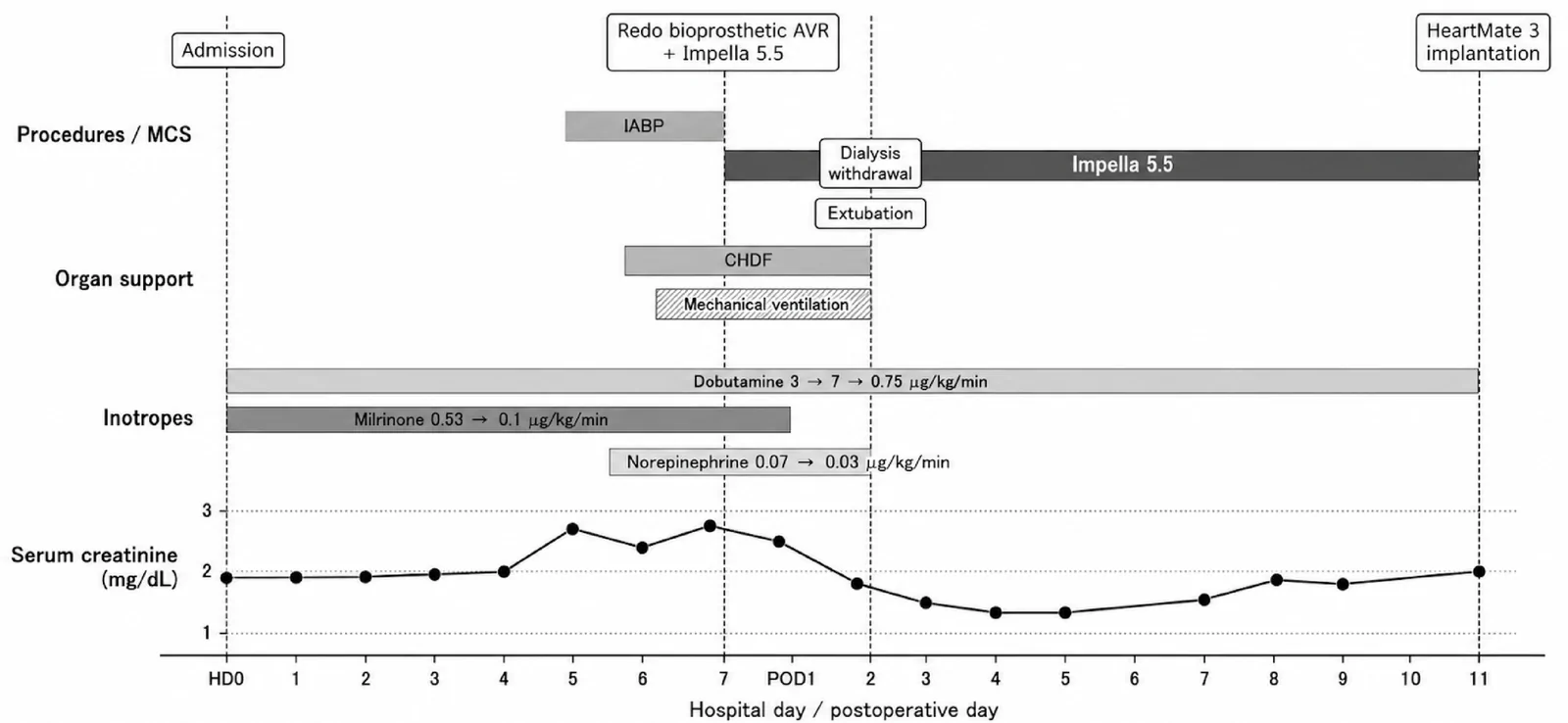
Clinical course of the staged treatment strategy. The figure summarizes the staged management from mechanical aortic valve replacement to redo aortic valve replacement with bioprosthetic valve conversion, Impella 5.5 support, and subsequent HeartMate 3 implantation. AVR, aortic valve replacement; HM3, HeartMate 3; LVAD, left ventricular assist device; MCS, mechanical circulatory support

Echocardiographic evaluation at the time of HeartMate 3 implantation revealed no structural deformation of the bioprosthetic leaflets, with only trivial central aortic regurgitation. This finding remained unchanged throughout the postoperative follow-up period. Under continuous-flow LVAD support, the bioprosthetic aortic valve remained closed throughout both systole and diastole. The overall clinical course is summarized in Fig. 3. The patient was discharged home in stable condition.

## Discussion

Cardiogenic shock after mechanical AVR is uncommon but presents a major therapeutic dilemma [5]. Mechanical valves prohibit transvalvular MCS, leaving extracorporeal support as the primary alternative. However, veno-arterial extracorporeal membrane oxygenation may exacerbate LV distension and increase complication rates [6]. These considerations suggest that prosthesis selection in young patients with severe aortic regurgitation and markedly reduced left ventricular function should not be based solely on age or valve durability. Although mechanical valves remain appropriate in many young patients, severely reduced preoperative left ventricular function may predict limited postoperative recovery and a future need for mechanical circulatory support. Therefore, in selected patients such as those with left ventricular EF < 25% and preoperative BNP 365 pg/mL, bioprosthetic aortic valve replacement may be a reasonable option to preserve the feasibility of future transvalvular support and durable left ventricular assist device strategies [1]. These findings suggest that prosthesis selection in young patients with severe aortic valve regurgitation and advanced LV dysfunction should be individualized by the Heart Team, with consideration of the potential future need for mechanical circulatory support.

Redo AVR with conversion to a bioprosthetic valve restores compatibility with transvalvular pumps, allowing effective LV unloading. The Impella 5.5 provides robust circulatory support and facilitates end-organ recovery, enabling safer transition to durable LVAD therapy [7, 8].

Although extracorporeal LVAD support can provide higher-flow temporary circulatory support, this strategy requires surgical LV apical cannulation, outflow graft placement, and management of an external circuit. If combined with valve conversion, the overall procedure would be substantially more complex than redo AVR alone. Furthermore, extracorporeal LVAD may be associated with significant complications including major bleeding, thromboembolic events, and infection, and a separate surgical procedure is typically required for device removal or conversion to durable LVAD therapy [9].

In contrast, the staged approach we adopted—redo bioprosthetic AVR followed by axillary Impella 5.5 insertion—allowed each step to be performed with a focused surgical objective, minimizing per-procedure complexity. Critically, bioprosthetic valve conversion simultaneously addressed two goals: enabling transvalvular Impella support and eliminating the need for any further valve surgery at the time of HeartMate 3 implantation. These considerations led our Heart Team to select the staged AVR-first approach as the most appropriate strategy for this specific patient.

In this case, staged intervention transformed a prohibitively high-risk patient into an acceptable candidate for destination therapy. Redo AVR with conversion to a bioprosthetic valve enabled Impella 5.5 support as a staged bridge to successful durable LVAD implantation in a patient initially considered unsuitable for LVAD therapy.

This report has several limitations. First, it describes a single case, and the generalizability of this strategy remains uncertain. Second, this approach requires redo aortic valve replacement in a critically ill patient and should therefore be considered only in carefully selected patients at experienced centers. Finally, the long-term durability and broader applicability of this strategy require further evaluation.

## Conclusion

Staged conversion from a mechanical to a bioprosthetic aortic valve combined with temporary MCS may provide an effective bridge to durable LVAD implantation in selected patients with cardiogenic shock after mechanical AVR. Strategic valve selection and individualized planning are essential in patients with advanced heart failure.

## Data Availability

Data sharing is not applicable to this article as no datasets were generated or analyzed during the current case report. Generative AI tools were used solely for English language editing and manuscript organization. All scientific content, interpretation, and final approval were performed by the authors, who take full responsibility for the content of the manuscript.
